# Supplementary material for: Zero- to ultralow-field J-spectroscopy with a diamond magnetometer
Source: Commun Chem. 2026 Mar 6;9:123. doi: 10.1038/s42004-026-01962-3 (PMC12992718; doi:10.1038/s42004-026-01962-3)
Supplement: Supplementary file 2 — Supplementary file (PDF) [file 42004_2026_1962_MOESM2_ESM.pdf]

Zero- to ultralow-field J-spectroscopy with a diamond magnetometer

Muhib Omar,[1,2,3] Jingyan Xu,[1,2,3] Raphael Kircher,[1,2,3] Pouya Sharbati,[1,2,3] Shaowen Zhang,[1,2,3] Georgios Chatzidrosos,[1,2,3] James Eills,[4] Román Picazo-Frutos,[1,2,3] Dmitry Budker,[1,2,3,5] Danila A. Barskiy,[1,2,3] and Arne Wickenbrock[1,2,3]

[1] *Johannes Gutenberg-Universität Mainz,55122 Mainz, Germany*
[2] *Helmholtz-Institut Mainz, 55128 Mainz, Germany*
[3] *GSI Helmholtzzentrum für Schwerionenforschung GmbH, 64291 Darmstadt, Germany*
[4] *Institute of Biological Information Processing (IBI-7), Forschungszentrum Jülich, 52425 Jülich, Germany*
[5] *Department of Physics, University of California, Berkeley, California 94720, USA*

(Dated: February 15, 2026)

A. Diamond sensor design choices

Pictures of the sensor head and the arrangement inside the magnetic shield can be seen in Fig. 2 and the most important components of the sensor head are detailed in Fig. 3. The diamond optics used for the collection of light are described in detail in reference[?]. The microwave (MW) and photodiode printed circuit board (PCB) designs are depicted in Fig. 1. The flexible PCB for the MW delivery has a thickness of $\approx 150\,\mu$m and a center hole for the sensing diamond to stick out by approximately $\approx 100\,\mu$m. The microwave board design was chosen such that circularly polarized microwaves could be applied. The photodiode PCB was designed to maximize compactness and avoid components that could generate residual magnetic fields. The photodiodes are mostly nonmagnetic (Hamamatsu S13228-01). Titanium screws (TI AL GmbH) were used to fasten the aluminum components. The fiber holder and the optics-holder rod were manufactured from titanium and aluminum, respectively.

The polycrystalline diamond plate was custom-fabricated by Medidia Tec GmbH and assembled with the other diamond components, the science diamond [Element Six (UK) Ltd.] and the diamond anvil (Dutch Diamond Technologies BV) at their facility. The sensing diamond was fixed to the diamond anvil using Norland Optical Adhesive 170.

The high-power optical fiber for light delivery (HPUCO-TA3AHPC-532-P-11AS) was purchased from OZ Optics Ltd., and any additional mounts for optics were 3D printed in-house.

B. Residual magnetic field of the diamond sensor components

To investigate possible sources of magnetic field (gradient) suspected to be responsible for the distortions of the spectra seen in Fig. ?? we measure ZULF NMR spectra with diamond sensor components placed next to the NMR sample. We used the same geometry as in Fig. ?? b). We place the components on a ceramic stick to move them to the closest position with respect to the NMR tube. The fiber and fiber holder components are tested outside the first layer of the magnetic shield, i.e., at their position during the NV ZULF-NMR measurement.

We record ZULF NMR spectra for each component with the OPM (see Fig. 4) and find no broadening in the spectrum with just the aluminum holder. Adding the diamond components broadens the linewidth in the 1J-peak by about 70%. Assembling the full sensor does not further broaden the spectrum. This indicates that the magnetic field gradients likely responsible for the broadening seem to be dominated by the diamond plate. The plate includes the NV diamond for sensing glued to the diamond anvil. We suspect the gradient could be related to the optical adhesive used to affix the NV diamond to the diamond optics or to the reflective coating applied on the sensing diamond. These cannot be studied separately with the current sensor but will be investigated in the future.

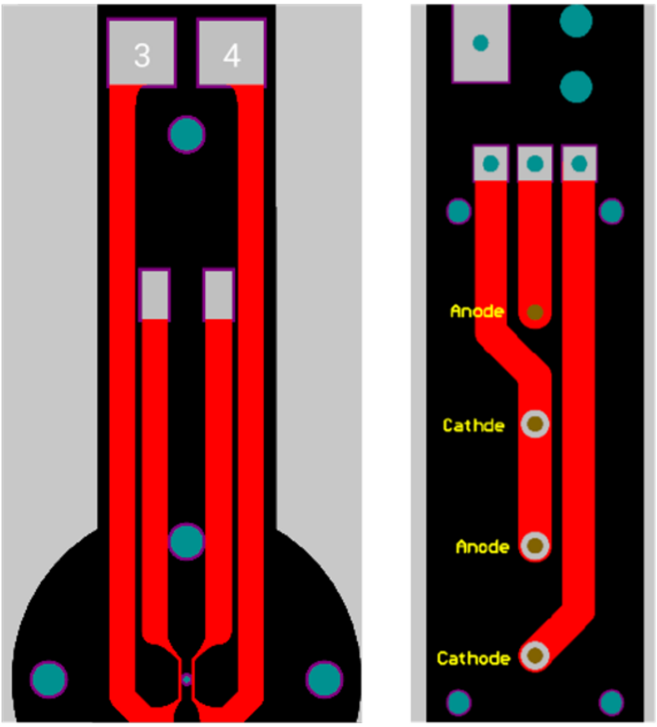

Supplementary Figure 1: Microwave delivery PCB (left). Photodiode assembly PCB (right).

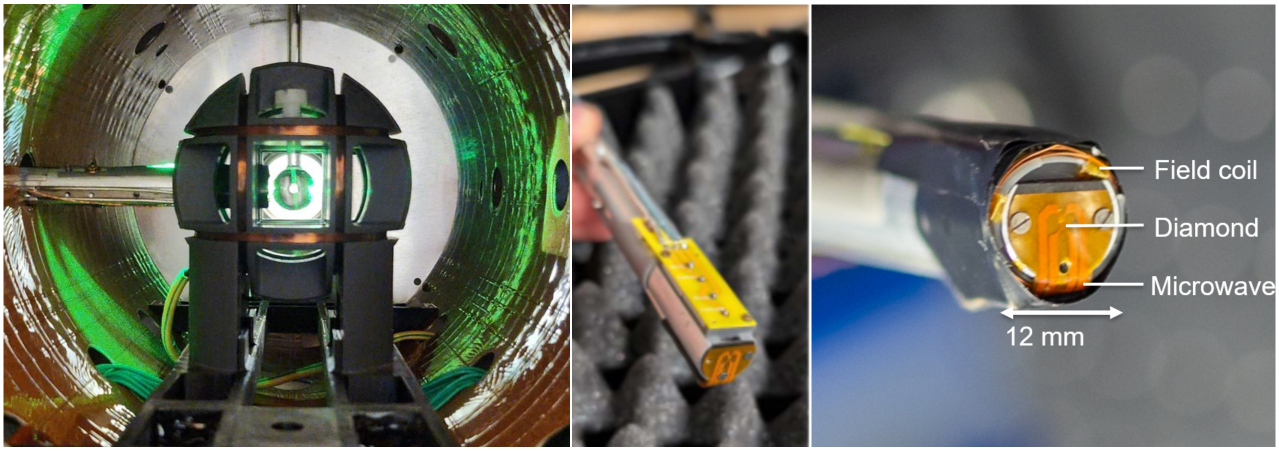

Supplementary Figure 2: Picture of the sensor head used for diamond ZULF NMR detection with the NMR sample within the MS2 magnetic shield (left). Picture of the diamond sensor assembly (middle) and close-up of the diamond sensor with annotations (right).

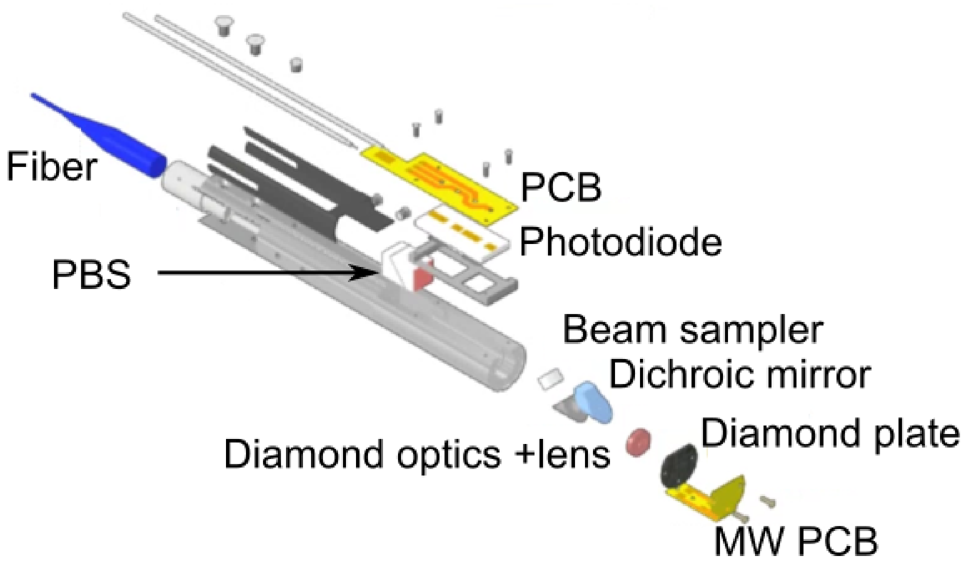

Supplementary Figure 3: Explosion view of the diamond sensor. PBS : polarizing beam splitter, PCB : printed circuit board, MW : microwave.

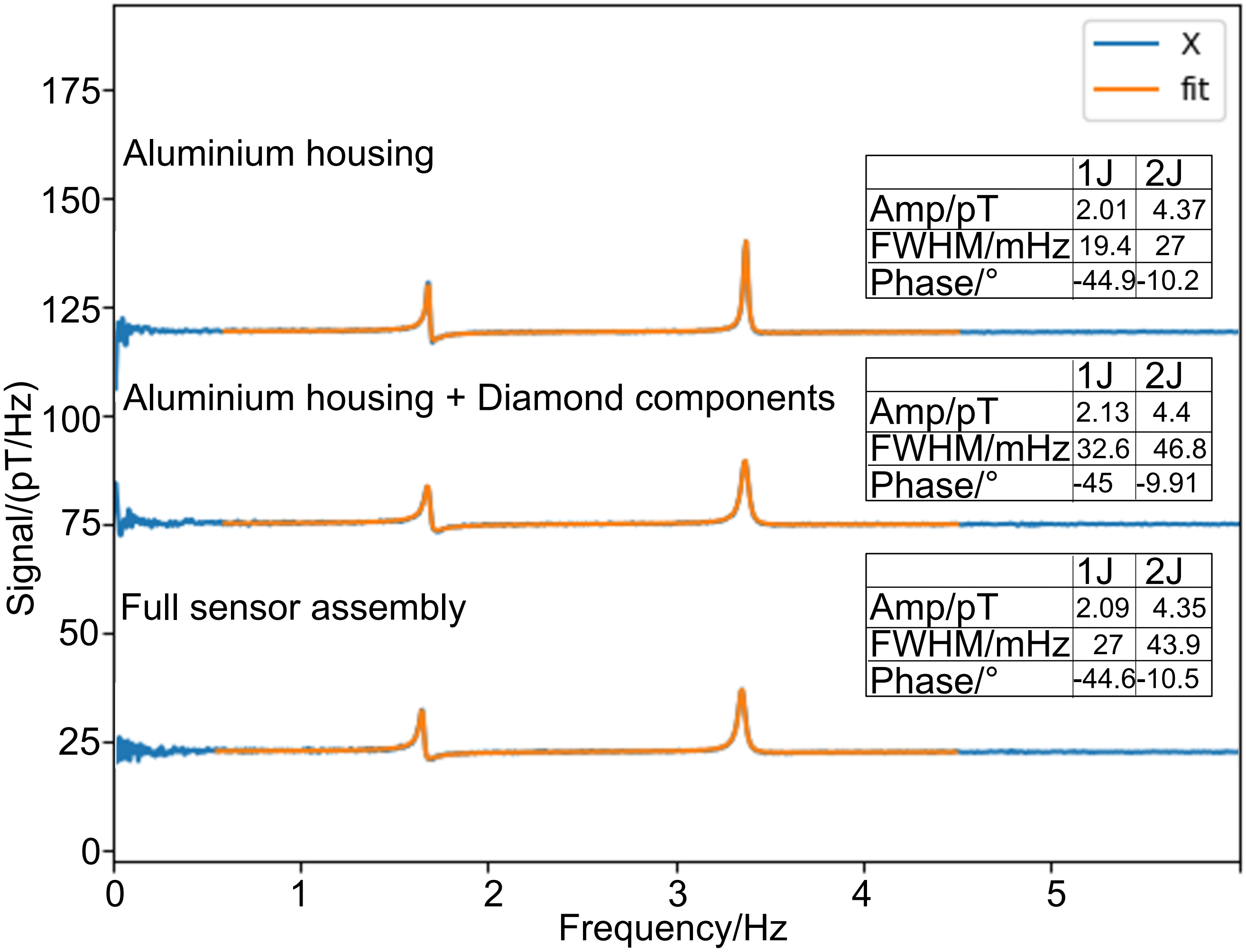

Supplementary Figure 4: Effect of diamond-sensor components on ZULF spectra. The spectra (real part of the FFT) are recorded with the OPM at a distance of 12 mm and fitted with two complex Lorentzians. The fit parameters are listed in the inset tables for both J peaks. The assembly with different diamond-sensor components are placed as close as possible to the sample cell (6.8 mm distance to the center). Broadening of the spectra are observable when adding the diamond components. For visual clarity, the spectra are displayed with vertical offsets. The fitting function is $L(f; f_0, Amp, \gamma, phase) = \frac{Amp}{\gamma^2/4+(f-f_0)^2}\left[\frac{\gamma}{2}\cos(phase) + (f-f_0)\sin(phase)\right]$, with Amp the amplitude, f the frequency, f_0 the center frequency per peak, 2γ the full width at half maximum (FWHM) and $phase$ the complex phase.
